# Supplementary material for: A fault-intrusion-tolerant system and deadline-aware algorithm for scheduling scientific workflow in the cloud
Source: PeerJ Comput Sci. 2021 Nov 2;7:e747. doi: 10.7717/peerj-cs.747 (PMC8576554; doi:10.7717/peerj-cs.747)
Supplement: Supplemental Information 6 [file peerj-cs-07-747-s006.zip › Myproject1.1/docs/org/cloudbus/cloudsim/UtilizationModelFull.html]

UtilizationModelFull


JavaScript is disabled on your browser.


- Overview
- Package
- Class
- Use
- Tree
- Deprecated
- Index
- Help

- Prev Class
- Next Class

- Frames
- No Frames

- All Classes

- Summary:
- Nested |
- Field |
- Constr |
- Method

- Detail:
- Field |
- Constr |
- Method


org.cloudbus.cloudsim

## Class UtilizationModelFull

- java.lang.Object
- - org.cloudbus.cloudsim.UtilizationModelFull

- All Implemented Interfaces:
  :   UtilizationModel

  ---

    

  ```
  public class UtilizationModelFull
  extends java.lang.Object
  implements UtilizationModel
  ```

  The UtilizationModelFull class is a simple model, according to which a Cloudlet always utilize
  all the available CPU capacity.

  Since:
  :   CloudSim Toolkit 2.0

- - ### Constructor Summary

    Constructors

    Constructor and Description

    `UtilizationModelFull()`
  - ### Method Summary

    Methods

    | Modifier and Type | Method and Description |
    | --- | --- |
    | `double` | `getUtilization(double time)` Returns utilization in percents according to the time. |

    - ### Methods inherited from class java.lang.Object

      `clone, equals, finalize, getClass, hashCode, notify, notifyAll, toString, wait, wait, wait`

- - ### Constructor Detail


    - #### UtilizationModelFull

      ```
      public UtilizationModelFull()
      ```
  - ### Method Detail


    - #### getUtilization

      ```
      public double getUtilization(double time)
      ```

      **Description copied from interface: `UtilizationModel`**

      Returns utilization in percents according to the time.

      **Specified by:**
      :   `getUtilization` in interface `UtilizationModel`

      Parameters:
      :   `time` - the time

      Returns:
      :   utilization percentage


- Overview
- Package
- Class
- Use
- Tree
- Deprecated
- Index
- Help

- Prev Class
- Next Class

- Frames
- No Frames

- All Classes

- Summary:
- Nested |
- Field |
- Constr |
- Method

- Detail:
- Field |
- Constr |
- Method
